# Resistance to bacteriocin Lcn972 improves oxygen tolerance of *Lactococcus lactis* IPLA947 without compromising its performance as a dairy starter

**DOI:** 10.1186/s12866-018-1222-8

**Published:** 2018-07-20

**Authors:** María Jesús López-González, Ana Belén Campelo, Antonia Picon, Ana Rodríguez, Beatriz Martínez

**Affiliations:** 1 Dairy Safe group, Department of Technology and Biotechnology of Dairy Products, Instituto de Productos Lácteos de Asturias, IPLA-CSIC, Paseo Río Linares, s/n 33300 Villaviciosa, Asturias Spain; 2 Departamento de Tecnología de Alimentos, Instituto Nacional de Investigación y Tecnología Agraria y Alimentaria (INIA), Carretera de La Coruña Km 7.5, 28040 Madrid, Spain

**Keywords:** Dairy starter, *Lactococcus lactis*, Bacteriocin, Oxidative stress, Adaptive evolution, Lcn972

## Abstract

**Background:**

*Lactococcus lactis* is the main component of the mesophilic starters used in cheese manufacture. The success of milk fermentation relies on the viability and metabolic activity of the starter bacteria. Therefore, robust strains able to withstand the harsh conditions encountered during cheese manufacture and starter production are demanded. In this work, we have applied adaptive evolution under cell envelope stress imposed by the cell wall active bacteriocin Lcn972 to evolve strains with more robust phenotypes.

**Results:**

Consecutive exposure of the starter strain *L. lactis* IPLA947 to Lcn972 yielded a stable mutant, *L. lactis* R5, with enhanced survival when challenged with hydrogen peroxide. *L. lactis* R5 exhibited faster growth rates in aerobic fermentations in broth and was able to acidify milk to a lower pH in aerated milk cultures. The improved behavior of *L. lactis* R5 in the presence of oxygen did not translate into a better performance in the presence of heme (i.e. respiration metabolism) or into higher survival during storage at cold temperatures or after freeze-drying compared to the wild type *L. lactis* IPLA947. *L. lactis* R5 retained the same milk acidification rate and no changes in the consumption of lactose and production of organic acids were noticed. However, the profile of volatile compounds revealed a significant increase in 3-hydroxy-2-butanone (acetoin) in curds manufactured with *L. lactis* R5.

**Conclusions:**

Based on our results, *L. lactis* R5 can be proposed as a suitable dairy starter with improved survival under oxidative stress and enhanced metabolic traits. The results support the notion that adaptive evolution under cell envelope stress might be useful to generate strain diversity within industrial *L. lactis* strains.

## Background

Lactic acid bacteria (LAB) are the main components of dairy starters and are used to produce a wide variety of fermented products such as cheese, butter, fermented milk, yogurt, etc., which are highly appreciated by consumers. Among LAB, *Lactococcus lactis* is the main component of the mesophilic starters used in cheese manufacture. The main function of *L. lactis* is to produce L (+) lactic acid from lactose leading to the pH decrease required for milk clotting. The enzymatic pool of *L. lactis* is further involved in the development of the flavor and texture of the final product [35]. For this reason, starter strains have been carefully selected according to their metabolic and technological properties along with the absence of undesirable traits (e.g. antibiotic resistance genes) [15]. Suitability for large scale production is also a major criterion in starter selection. A key step forward has been achieved by the introduction of a starter production strategy based on respiration (revised by [28]). Biomass yields and survival of *Lactococcus* and other LAB are greatly enhanced by aerobic growth in the presence of heme, i.e. under respiration metabolism where oxygen is reduced to water and protons are extruded [14, 30]. Otherwise, the presence of oxygen originates reactive oxygen species that compromise *L. lactis* growth [7]. Indeed, several studies have confirmed that dissolved oxygen, as well as the positive redox potential of milk, can seriously compromise the acidification rate of *L. lactis* in milk [16, 22].

Considering that the success of milk fermentation relies on the viability and metabolic activity of *L. lactis*, continuous efforts have been made to understand the physiology behind robust phenotypes of industrially relevant microorganisms (reviewed by [27]). The knowledge gained on *L. lactis* stress physiology has been translated, mostly by recombinant DNA technology, into engineered strains with superior traits. However, legal constraints and the negative opinion of consumers towards genetically modified organisms (GMOs) pose a barrier for further marketing and create demand for alternative strategies [12, 20].

Adaptive evolution is becoming a popular non-GMO strategy to enhance starter fitness and develop evolved strains with enhanced technological traits. During adaptive evolution, bacterial populations are confronted with a particular stressor for several generations to select for mutations that enhance fitness under those prevailing conditions [37]. Adaptive evolution has been recently applied, for example, to improve *L. lactis* tolerance to high temperature [8] and acid tolerance in *Lactobacillus casei* and *Leuconostoc mesenteroides* [17, 38]. Interestingly, a common occurrence in adaptive evolution experiments is the selection of additional phenotypes not directly related to the applied stress, and which may likely arise due to mutations in global regulators, metabolic adaptations and/or cross-protection phenomena [8, 27].

The aim of this work was to assess adaptive evolution under cell envelope stress as a means to enhance robustness of *L. lactis*. Cell envelope stress was applied by exposure to the anti-lactococcal bacteriocin Lcn972 that inhibits cell wall biosynthesis by specifically binding to the cell wall precursor lipid II, thereby triggering the cell envelope stress response in *L. lactis* [24, 25]. Previous work carried out with the laboratory strain *L. lactis* MG1614 (a derivative of the laboratory strain *L. lactis* MG1363) suggested that exposure to Lcn972 activated genes that contribute to survival to heat and acid stresses [31]. Moreover, Lcn972-resistant mutants of *L. lactis* MG1614 had a more densely packed peptidoglycan and increased resistance to nisin and lysozyme [32]. Based on these observations, we hypothesized that continuous exposure of *L. lactis* to Lcn972 might result in evolved strains with additional beneficial traits, besides becoming resistant to Lcn972. We have applied this strategy to the industrial strain *L. lactis* IPLA947 (CECT 5180), which is the main acidifying strain in a mixed-strain starter culture designed for the manufacture of the Afuega’l pitu cheese [3]. This cheese is an acid-coagulated cheese, made with pasteurized milk, in which coagulation relies heavily on the optimal activity of the added starter bacteria [10]. Because the initial levels of oxygen in milk may interfere with acidification by *L. lactis*, we have specifically studied if evolved strains could better withstand oxidative stress.

## Methods

### Bacterial strains and growth conditions

*L. lactis* IPLA947 [3] was incubated at 30 °C in M17 (Biokar Diagnostics, Beauvais, France) containing lactose (0.5%) as a carbon source. Batch cultures were carried out in standing 12-ml tubes filled in with 10 ml of M17 broth. Aerated cultures were grown in 50-ml Falcon tubes with culture volumes not exceeding 20 ml and under shaking at 250 rpm. When required, hemin (Sigma-Aldrich, Alcobendas, Spain) was added to M17 at 0.01 mg/ml to activate respiration. Growth parameters were calculated from, at least, two independent cultures started by inoculating pre-warmed M17 with overnight cultures at an optical density at 600 nm (OD_600_) of 0.05. Growth rates (μ) and generation times (g) were calculated by linear regression of ln(OD_600_) versus time in the exponential phase and as ln(2)/μ, respectively. OD_600_ measurements were done in 1-cm cuvettes in a Biophotometer (Eppendorf, Hamburg, Germany) or followed in a Benchmark Plus Microplate spectrophotometer (BioRad Laboratories, Hercules, CA, USA), as indicated. Colony forming units per milliliter (CFU/ml) were determined by plating serial decimal dilutions made in Ringer solution on M17 agar plates (Merck, Darmstadt, Germany).

### Adaptation to Lcn972 and susceptibility tests

Lcn972 was purified and quantified as described elsewhere [25] and stocks (320 μg/ml, 12,800 AU/ml) were kept at − 20 °C in 50 mM sodium phosphate buffer, pH 6.8. Adaptation was approached as previously described [32] with modifications. *L. lactis* IPLA947 was inoculated at 1% (*v*/v) from an overnight culture and was sequentially cultivated at 30 °C for 16 h in M17 (2 ml) with doubling Lcn972 concentrations ranging from 20 to 1280 AU/ml. Decimal dilutions from cultures grown with 1280 AU/ml were prepared in Ringer solution and plated on M17 agar to isolate single colonies. Selected colonies were consecutively grown in M17 for 100 generations in the absence of Lcn972. After colony purification, stabilized cultures were stored at − 80 °C in M17 broth supplemented with 10% glycerol for further characterization. Minimal inhibitory concentrations (MICs) were determined during the stabilization step (i.e. growth without Lcn972) by the broth microdilution method [24]. To do that, overnight cultures were first adjusted to an OD_600_ of 0.5 and then further diluted 1:1000 to prepare a cell suspension with a concentration of 3 × 10^5^ CFU/ml, which was subsequently used to inoculate microtiter plates, containing a gradient of Lcn972 concentrations.

### Resistance to hydrogen peroxide

Survival to oxidative stress was determined according to Dijkstra et al. [13] with some modifications. Briefly, overnight M17 cultures were resuspended in 50 mM sodium phosphate buffer, pH 6.8, and exposed to 5 mM H_2_O_2_ for 2 h at 30 °C in a water bath. Decimal dilutions were plated on M17 agar plates. Untreated controls were handled equally but using sodium phosphate buffer instead of H_2_O_2_. Three independent cultures per strain were analyzed.

### Phase-contrast microscopy

Aliquots of exponentially (4 h) and stationary (24 h) cultures were deposited on glass slides and observed with a DMi8 inverted microscope (Leica microsystems, Wetzlar, Germany). Cell length and width was measured in cells (*n* = 32) from four different fields per sample using the microscope built-in software LasX.

### Survival during cold storage

Late exponentially growing cultures of *L. lactis* IPLA947 and R5 were used to inoculate 20 ml of M17 at OD_600_ 0.05. Subsequently, 10-ml aliquots were transferred to 12-ml tubes for batch incubation at 30 °C or to 50-ml tubes which were agitated at 250 rpm. Incubation proceeded for 14 h (early stationary phase) before storage at 4 °C. Samples were taken at weekly intervals and viability (CFU/ml) was determined by plating decimal dilutions on M17 agar plates. Two independent cultures per strain were analyzed.

### Lyophilisation

Cells from late exponentially growing batch cultures (100 ml) of *L. lactis* IPLA947 and R5 were collected, washed with Ringer solution and resuspended in 10 ml of 11% reconstituted Difco skim milk (Becton Dickinson, Franklin Lakes, NJ, USA). Samples were frozen at − 80 °C and lyophilized in a Virtis Freezemobile 12EL (VirTis, Gardiner, NY, USA) for 48 h. Lyophilized powder was homogenized by vigorous shaking and stored in 50-ml tubes at 4 °C.

### Milk acidification and detection of lactose and organic acids

Milk cultures were carried out in commercially available homogenized UHT milk (CAPSA, Granda, Spain). Milk (50 ml) was inoculated at 2% (*v*/v) with M17 overnight cultures, which were previously washed twice with Ringer solution, and then incubated at 21 °C for 19 h. Acidification was monitored with a real-time pHmeter ORION™ Versa Star™ (Thermo Scientific Inc., Waltham, MA, USA), which recorded pH values every 15 min. As descriptors of the acidification curves, we use maximum acidification rates (Vm), defined as pH decrease (mU) per min, the time interval at which the maximum acidification rate was maintained (Tm), as well as the time in min (Te) to reach pH 4.6 [19]. Curves were performed in duplicate.

At the end of the incubation, samples (1 ml) were homogenized with 5 ml of 4.5 mM H_2_SO_4_ for 1 h at 37 °C under continuous shaking, centrifuged (16,000 x g, 15 min, 4 °C), and filtered (0.22 μm). Lactose and organic acids were determined by HPLC using an ICSep ICE-ION-300 ion-exchange column (mobile phase 0.0085 N H_2_SO_4_, operating temperature 65 °C, flow rate 0.4 ml min^− 1^). A 996 Photodiode Array Detector (Waters) for the determination of organic acids (detection wavelength 210 nm), and a Waters 410 differential refractometer for sugar determination (detection wavelength 280 nm) were used. For quantification, regression equations (R^2^ ≥ 0.99) were calculated by using different concentrations of the corresponding standards.

### Detection of volatile compounds

*L. lactis* IPLA947 and R5 were activated by two successive transfers in 10% reconstituted skim milk prior to inoculation at 1% (*v*/v) in fresh semi-skim pasteurized milk supplemented with 1 ml l^− 1^ of a 100 g l^− 1^ CaCl_2_ solution. They were grown in two independent experiments at 25 °C for 24 h in 100 ml glass flasks. Cultures were transferred, with the help of a sterile spatula, to centrifuge tubes and centrifuged at 12857 x g for 20 min in an Eppendorf 5810R centrifuge (Eppendorf, Hamburg, Germany). Pellets were wrapped in aluminium foil, vacuum packed in HT3050 plastic bags (Cryovac Sealed Air Corporation, Milano, Italy) and kept at − 35 °C until further analysis. Duplicate curd samples (7 g) were homogenized in a mechanical grinder with 14 g of anhydrous Na_2_SO_4_ (Merck, Darmstadt, Germany) and 25 μl of an aqueous solution of 495 mg l^− 1^ cyclohexanone (Sigma-Aldrich) added as internal standard. Volatile compounds were extracted by solid-phase microextraction (SPME) using a Divinylbenzene/Carboxen/Polydimethylsiloxane (DVB/CAR/PDMS) coated fibre (Supelco, Bellefonte, PA, USA). They were analyzed and identified by gas chromatography–mass spectrometry (GC–MS) (HP 6890-MSD HP 5973, Agilent, Palo Alto, CA, USA) as previously described [29].

### Statistical analyses

Statistically significant differences were analyzed by Student t-test as implemented in Microsoft Excel 2010 (Microsoft Corporation). One-way analysis of variance was conducted with IBM SPSS Statistics (ver. 24.0.0.1) to compare cell length and width of IPLA947 and R5 cells from aerobic and batch cultures. A *P* value below 0.05 was considered statistically significant.

## Results and discussion

We have explored exposure of a *L. lactis* dairy starter strain to the cell wall active bacteriocin Lcn972 as a means of obtaining evolved strains with robust phenotypes. The reasoning behind this was that resistance to Lcn972 in laboratory strains is accomplished through several mechanisms that, as a whole, contribute to enhanced survival under harsh conditions. Such mechanisms involved remodeling of the cell envelope towards a more densely packed peptidoglycan, the likely production of structural or surface polysaccharides and the activation of genes with protecting functions [1, 32, 33]. However, it was not possible to anticipate if similar results could be obtained with industrial or environmental *L. lactis* strains because they often differ phenotypically and genetically from laboratory strains [6, 18]. Therefore, as a proof of concept, we applied this strategy to the starter strain *L. lactis* IPLA947.

### Selection of *L. lactis* IPLA947 derivatives resistant to Lcn972

The MIC of the bacteriocin Lcn972 for *L. lactis* IPLA947 was 20 AU/ml (Table 1). This value was similar to those described for other lactococcal strains isolated from commercial dairy starters and for the laboratory strain *L. lactis* MG1614 [23]. Adaptive evolution under cell envelope stress (CES) was conducted by a two-step process consisting of an adaptation step, which involved exposing the cultures to increasing amounts of Lcn972, and a subsequent stabilization step, in which bacterial cultures were grown for 100 generations in the absence of Lcn972. Adapted cultures were able to grow at bacteriocin concentrations of up to 1280 AU/ml, which is 64 times the initial MIC and near the immunity levels (1600 AU/ml) provided by the Lcn972 immunity proteins in Lcn972 producers [2]. Higher concentrations inhibited growth and fully dense M17 cultures (i.e. OD_600_ = 2.0–2.5) could not be reached in 16 h. The culture able to grow at 1280 AU/ml was serially diluted to isolate single colonies and twenty-two colonies were randomly selected. Their MICs ranged from > 160 AU/ml (9 clones), 160 AU/ml (12 clones) to 80 AU/ml (1 clone) (data not shown). Three resistant variants, with MICs over 160 AU/ml (R2A and R3A) and 80 AU/ml (R5A), were selected for further characterization. Partial sequencing of the 16S rDNA and RAPD-PCR profiles confirmed the identity of these clones and excluded unintentional contamination (data not shown).

**Table 1:** Properties of *L. lactis* IPLA947 and its Lcn972-resistant derivatives R2, R3 and R5

*L. lactis*	MIC Lcn972 (AU/ml)	Growth rate (μ) and generation time (g)^a^					
		Batch		Aeration^b^		Respiration	
		μ (h^−1^)	g(h)	μ (h^−1^)	g(h)	μ (h^−1^)	g(h)
947 (WT)	20	0.64 ± 0.10	1.10 ± 0.16	0.38 ± 0.02	1.81 ± 0.08	0.86 ± 0.01	0.81 ± 0.01
R2	> 160	0.60 ± 0.02	1.16 ± 0.05	0.43 ± 0.01*	1.62 ± 0.04	ND	ND
R3	> 160	0.63 ± 0.02	1.11 ± 0.03	0.44 ± 0.01*	1.59 ± 0.04	ND	ND
R5	80	0.63 ± 0.01	1.10 ± 0.02	0.50 ± 0.01**	1.38 ± 0.04*	0.84 ± 0.01	0.83 ± 0.01

^a^Two independent cultures were analysed. Results are expressed as means ± standard deviation

^b^Significantly different from the wildtype (WT) *L. lactis* IPLA947. (*) *P* < 0.05; (**) *P* < 0.01

*ND*: not determined

After adaptation, a single colony from each resistant variant was consecutively grown in M17 in the absence of selective pressure, to confirm that the higher Lcn972 MICs shown by these evolved clones was not transitory, i.e. as a result of the induction of the CES response orchestrated by the two-component system CesSR [25]. Contrary to our previous observation with the laboratory strain *L. lactis* MG1614 [32], the resistant phenotype of the selected clones was not lost during stabilization and they retained the same Lcn972 MIC recorded right after the adaptation step (“RnA” strains) (Table 1). From each stabilized culture of R2A, R3A and R5A, a single colony was stored and named R2, R3 and R5, respectively. To assess possible fitness costs associated to the Lcn972 resistant phenotype, growth of *L. lactis* IPLA947 and its Lcn972R derivatives in M17 was monitored in a microtiter plate reader at 30 °C (Table 1). All the resistant strains had similar growth rates and no significant differences were noted (*P* > 0.05) compared to the wildtype strain. Therefore, stable Lcn972 resistant mutants derived from this industrial strain could be isolated and the putative mutations accumulated during adaptive evolution did not seem to pose a physiological burden.

### Resistance to hydrogen peroxide

**Fig. 1:**
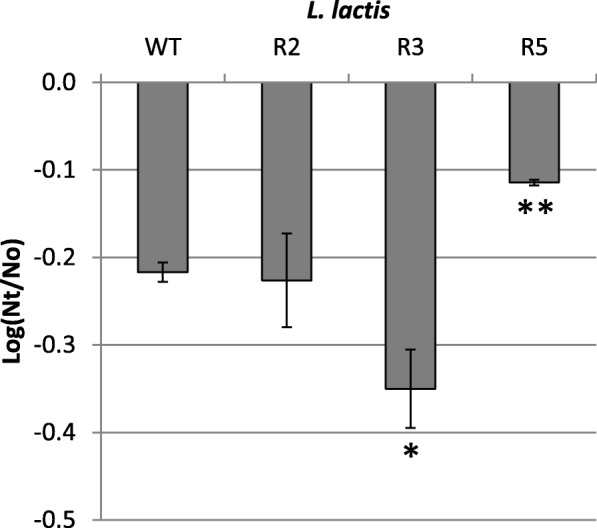
Survival of *L. lactis* IPLA947 (WT) and its Lcn972R derivatives *L. lactis* R2, R3 and R5 after exposure to 5 mM H_2_O_2_ for 2 h at 30 °C. Nt: CFU/ml after treatment; No: CFU/ml control. Results are the average of three independent cultures. Error bars are standard deviations. (*) *P* < 0.05; (**) *P* < 0.01, significantly different from *L. lactis* IPLA947

To initially address if the Lcn972R mutants had become resistant to oxidative stress, their viability was measured after being challenged with 5 mM H_2_O_2_ (Fig. 1). Under these conditions, *L. lactis* R2 behaved as the wildtype strain, while *L. lactis* R3 was the most sensitive with a viability loss of 0.4 Log CFU/ml. In contrast, the viability loss of mutant *L. lactis* R5 was significantly reduced by 50% (*P* < 0.01) compared to the wildtype strain, supporting the notion that *L. lactis* R5 coped with oxidative stress better after adaptive evolution under CES. These results anticipate the likely selection for different mutations within the population during adaptive evolution which is further supported by the different Lcn972 MICs (see Table 1).

### Growth of Lcn972 resistant derivatives in the presence of oxygen

Resistance to oxidative stress was evaluated in aerated M17 cultures as well. Faster growth rates and reduced generation times (Table 1) were observed for all Lcn972R mutants over those of the wildtype strain. *L. lactis* R2 and R3 grew moderately faster (*P* < 0.05) than the wildtype strain but more slowly than *L. lactis* R5. In particular, the *L. lactis* R5 strain showed a 32% improvement in growth rate.

Prompted by this result and the higher resistance of *L. lactis* R5 to H_2_O_2_, this mutant was further characterized. The performance of *L. lactis* R5 under respiration conditions (i.e. in the presence of hemin) was compared to that of the wildtype *L. lactis* IPLA947. In agreement with literature reports [28], both *L. lactis* IPLA947 and R5 grew faster under respiration conditions than in aerated cultures, almost doubling the growth rate (Table 1). However, the enhanced growth of the evolved *L. lactis* R5 in aerated cultures did not translate into a better performance under respiration as no significant differences in growth rate were observed between R5 and IPLA947 (Table 1). It has been reported that respiring *lactococci* are under lower oxidative stress due to the elimination of oxygen by the respiration metabolism [30]. Therefore, it is possible that the enhanced tolerance of *L. lactis* R5 to oxidative stress is masked under respiration, i.e. the mutations acquired during adaptive evolution may not represent an advantage under respiration conditions.

**Fig. 2:**
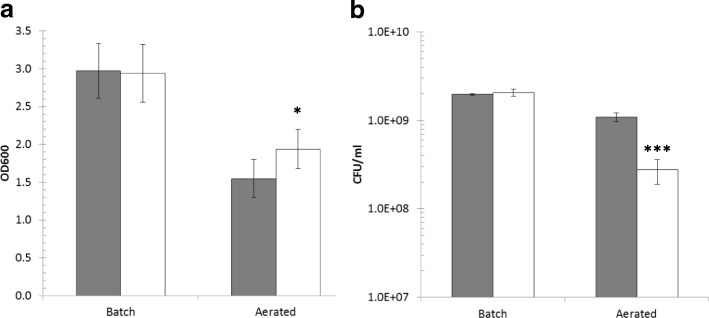
Optical density (OD_600_) (**a**) and CFU/ml (**b**) of batch and aerated cultures of *L. lactis* IPLA947 (grey bars) and the Lcn972R derivative *L. lactis* R5 (white bars) at the end of the exponential phase. Results are the average of two independent cultures. Error bars are standard deviations. (*) *P* < 0.05; (***) *P* < 0.001, significantly different from *L. lactis* IPLA947

**Fig. 3:**
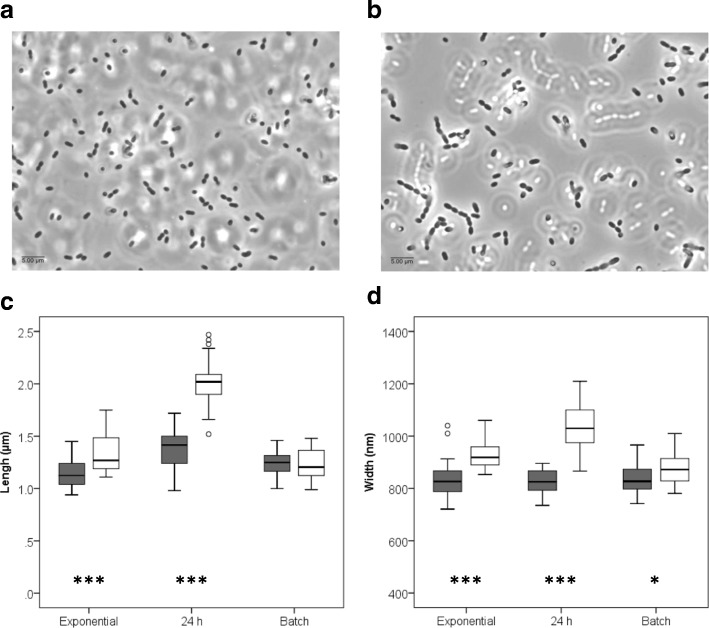
Cell morphology of *L. lactis* IPLA947 and the Lcn972R derivative *L. lactis* R5. Phase contrast microscopy of *L. lactis* IPLA 947 (**a**) and *L. lactis* R5 (**b**) sampled during growth in the presence of oxygen. Length (**c**) and width (**d**) of cells of *L. lactis* IPLA947 (grey) and *L. lactis* R5 (white) from exponentially and stationary (24 h) aerated cultures and exponentially growing batch cultures. (*) *P* < 0.05; (***) *P* < 0.001, significantly different from *L. lactis* IPLA947

**Fig. 4:**
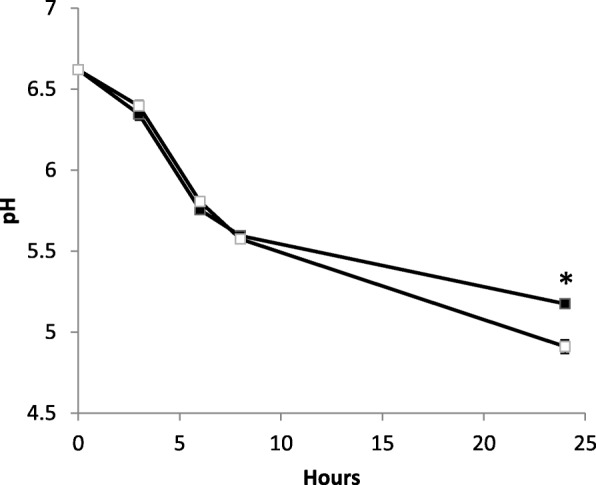
Decrease of pH in *L. lactis* IPLA947 (black squares) and the Lcn972R mutant *L. lactis* R5 (white squares) in aerated cultures in milk. Results are the average of two independent cultures. Error bars are standard deviations. (*) *P* < 0.05, significantly different from *L. lactis* IPLA947

An intriguing observation during these growth experiments was that, while in batch cultures *L. lactis* IPLA947 and R5 strains reached similar maximum OD_600_ values, in aerated cultures the evolved *L. lactis* R5 consistently reached higher OD_600_ than the wildtype strain (Fig. 2a). However, this increment in OD_600_ did not translate into higher CFU counts which were 0.6 Log units lower than the CFU counts of *L. lactis* IPLA947 (Fig. 2b). Discrepancies between OD_600_ values and CFU counts might be explained by changes in cell morphology, by the presence of longer chains which would underestimate cell counts as one chain would yield a single colony, or by a viable but non-culturable state [36]. Hence, new aerated cultures of *L. lactis* IPLA947 and R5 strains were started and samples were taken for microscopy observation during exponential (4 h) and stationary (24 h) growth. Two main morphological features were observed by phase-contrast microscopy (Fig. 3). On the one hand, *L. lactis* R5 formed longer chains than the wildtype strain in aerated cultures during the exponential phase (Fig. 3a, b). Also, R5 cells were both longer and thicker than those of the wildtype strain (Fig. 3c, d), particularly after prolonged incubation times. These differences were not observed in batch cultures (Fig. 3). According to these results, the lower CFU counts of *L. lactis* R5 seem to be due to the formation of longer chains and the higher OD_600_ values night be related to the increase in cell size. To further confirm the enhanced growth of *L. lactis* R5 in the presence of oxygen, aerated cultures of *L. lactis* IPLA947 and R5 were carried out in milk and the pH decrease was measured as an indication of metabolic activity. Milk was inoculated in duplicate and incubated at 30 °C with vigorous shaking. During the first 8 h, a similar trend was observed for both strains, but after 24 h, *L. lactis* R5 reached a significantly (*p* < 0.05) lower pH value than the wildtype strain (4.91 ± 0.04 vs 5.18 ± 0.02) (Fig. 4). This result confirmed that the evolved *L. lactis* R5 performed better than the wildtype strain in the presence of oxygen not only in M17 broth but also in milk. This enhanced trait could have important technological consequences because the presence of oxygen in milk due to milk handling in the factory may slow down the acidification rate [16, 22].

### Survival during cold storage

**Fig. 5:**
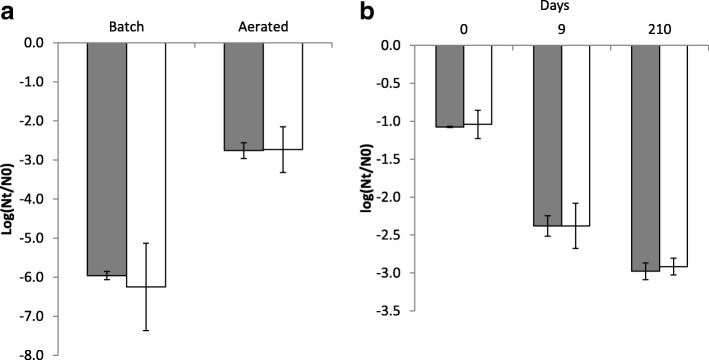
Survival during cold storage of *L. lactis* IPLA947 (grey) and the Lcn972R derivative *L. lactis* R5 (white). (**a**) Viability of batch and aerated cultures in M17 broth stored for 5 weeks at 4 °C. (**b**) Viability after freeze-drying and stored at 4 °C under air. Results are the average of two independent experiments. Error bars are standard deviations

We sought to determine if the better performance of *L. lactis* R5 strain during aerobic growth compared to the wildtype strain also implied better viability upon storage at refrigeration temperatures. Thus, *L. lactis* IPLA947 and R5 strains were either incubated statically or shaken for 14 h prior to storage at 4 °C and samples were subsequently taken on a weekly basis to check their viability. The initial bacterial counts remained constant for 3 weeks and decreased substantially after 5 weeks of storage (Fig. 5a). Survival of aerated cultures was roughly 3 Log units higher compared to batch cultures (*P* < 0.05), which is in line with previous reports showing that fermentation conditions may enhance *L. lactis* robustness and viability [13]. However, the R5 strain did not survive better than *L. lactis* IPLA947 (*P* > 0.05).

We also compared survival after freeze-drying and storage at 4 °C under air. Freeze-drying is a common method to preserve and deliver dairy starters, in which several factors including storage conditions are deemed relevant to maintain cell viability [4, 34]. For example, loss of membrane integrity due to oxidation of unsaturated fatty acids occurs during storage in the presence of oxygen, leading to reduced viability during storage [5]. As shown in Fig. 5b, the viability of *L. lactis* IPLA947 and R5 decreased during storage under air down to 3 Log units after 7 months but no differences were observed among them. Based on these results, the mutations acquired by *L. lactis* R5 seemed to protect it against exposure to oxygen during active growth but were not sufficient to guarantee better survival during storage.

### Performance of *L. lactis* R5 in milk fermentation

Growth in milk of *L. lactis* R5 was followed to verify that no major technological traits were lost upon adaptation to Lcn972 and that the evolved *L. lactis* R5 strain would still be as suitable as a dairy starter as the parental strain *L. lactis* IPLA947. The acidification rate was tested at 21 °C, the temperature at which Afuega’l Pitu cheese is coagulated [10]. As shown in Table 2, no significant differences (*P* > 0.05) were observed in any of the descriptors of the acidification curves. Similar maximum acidification rates were observed in both strains. The time to reach pH 4.6, the isoelectric point of casein, was extended by an average of 45 min in *L. lactis* R5 but the data were not significantly different (*P* > 0.05) (Table 2). The metabolic profile was also similar. After 19 h of incubation, residual lactose and production of the major organic acids (lactic, acetic and formic acids) were similar in both cultures (Table 2). Nonetheless, *L. lactis* R5 seemed to consume lactose more efficiently and showed a slightly higher mixed-acid metabolism compared to the wildtype strain, although these differences were not statistically significant (*P* > 0.05).

**Table 2:** Acidification and metabolites produced by *L. lactis* IPLA947 and the Lcn972 resistant mutant *L. lactis* R5 grown in milk

*L. lactis* strain	Acidification parameters^a^			Residual lactose (mM)	Organic acids (mM)		
	Vm (mpHUnits/min)	Tm (min)	Te (min)		Lactic	Acetic	Formic
947 (WT)	3.27 ± 0.04	150	960 ± 0	103.8 ± 6.5	61.8 ± 4.2	0.68 ± 0.07	0.27 ± 0.02
R5	3.32 ± 0.11	150	1005 ± 21	91.5 ± 4.9	52.4 ± 2.5	1.01 ± 0.02	0.24 ± 0.00

^a^*Vm* maximum acidification rate, *Tm* time interval at which the maximum acidification rate was maintained, *Te* time to reach pH 4.6. Results are expressed as means ± standard deviation of two independent experiments

**Fig. 6:**
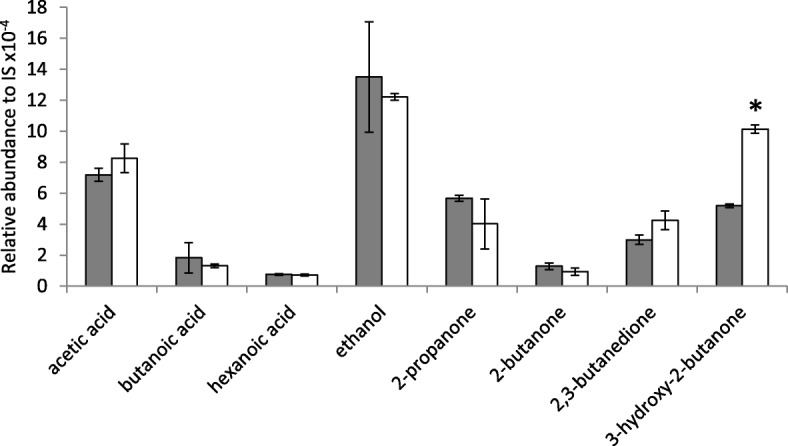
Volatile compounds detected by GC-MS in milk cultures of *L. lactis* IPLA947 (grey bars) and the Lcn972R mutant *L. lactis* R5 (white bars). Results are the mean ± standard deviation of two independent cultures. IS, internal standard (*) *P* < 0.05, significantly different from *L. lactis* IPLA947

In a separate experiment, the volatile profile of curds made with *L. lactis* IPLA 947 and R5 strains was determined. After 24 h of incubation, eight volatile compounds (three acids, one alcohol and four ketones) were detected. Levels of volatile compounds for both strains were not significantly (*P* < 0.05) different, with the only exception of 3-hydroxy-2-butanone (acetoin), whose levels were almost twofold higher in curds produced by *L. lactis* R5 than in those produced by *L. lactis* IPLA947 (Fig. 6). Acetoin is synthesized from pyruvate by the enzyme acetolactate synthase when the NADH pool is low and the activity of lactate and pyruvate dehydrogenases is not optimal [26]. The increased levels of acetoin in *L. lactis* R5 curd suggest activation of the acetolactate synthase pathway, diverting pyruvate metabolism towards acetoin production. This pathway has been shown to be activated to cope with the presence of oxygen in both M17 broth and milk cultures [9, 21]. It seems plausible that, due to the stress imposed by Lcn972 during adaptive evolution, metabolic adaptations might have occurred as a strategy to produce additional energy and lower the stress level, as often described in the literature [27]. Therefore, mutations leading to the activation of the acetolactate synthase pathway might be behind the enhanced response of *L. lactis* R5 to oxidative stress. Moreover, acetoin, the reduction product of 2,3-butanodione (diacetyl), imparts a sour-milk aroma and together with diacetyl, which has buttery and vanilla notes, are key aroma components of many cheese varieties [11]. Hence, the incorporation of the evolved *L. lactis* R5 strain as part of the Afuega’l pitu cheese starter might improve cheese flavor. Nevertheless, Afuega’l pitu cheese pilot plant trials using pasteurized milk should be carried out to confirm the performance of the evolved strain under production conditions.

## Conclusions

In this work, it has been shown that adaptive evolution of the industrial starter *L. lactis* IPLA947 under cell envelope stress may provide evolved strains with enhanced robustness phenotypes as exemplified by the higher tolerance to oxygen of *L. lactis* R5. Furthermore, adaptation to Lcn972 did not compromise essential technological parameters, such as milk acidification rate, and interesting metabolic changes were selected that might improve cheese flavour. Although it cannot be anticipated that every single *L. lactis* strain will evolve in a similar fashion as *L. lactis* IPLA947, our proposed strategy might be useful to generate strain diversity within industrial *L. lactis* strains, and could be adopted by the dairy industry in product development and market expansion.

The complexity of the stress-responsive regulatory networks that often overlap and provide cross-protection, together with additional metabolic adaptations, makes it difficult to predict and draw hypotheses on the putative mutations selected during adaptive evolution that are involved in resistance to the cell wall active Lcn972 and/or oxygen tolerance. Genome sequencing and transcriptional analyses will have to be approached in the near future to identify the molecular basis of the observed phenotypes and hopefully the results may contribute to a better understanding of the stress physiology of *L. lactis*.
